# S100 Calcium-Binding Protein A6 Promotes Epithelial-Mesenchymal Transition through β-Catenin in Pancreatic Cancer Cell Line

**DOI:** 10.1371/journal.pone.0121319

**Published:** 2015-03-23

**Authors:** Xue Chen, Xinjuan Liu, Haibo Lang, Shiqi Zhang, Yanlin Luo, Jie Zhang

**Affiliations:** 1 Department of Gastroenterology, Beijing An Zhen Hospital, Capital Medical University, Beijing, China; 2 Department of Gastroenterology, Beijing Chao-Yang Hospital, Capital Medical University, Beijing, China; 3 Department of neurobiology and Beijing institute for brain disorders, School of basic medical science, Capital Medical University, Beijing, China; National Institute for Viral Disease Control and Prevention, CDC, China, CHINA

## Abstract

The pathogenesis of pancreatic ductal adenocarcinoma (PDAC) remains poorly understood. S100 calcium-binding protein A6 (S100A6) has been associated with PDAC; however, the effect of S100A6 on PDAC migration and invasion has not yet been explored. In this study, Panc-1 cells were transfected with a plasmid to induce overexpression of S100A6, and β-catenin was knocked down using a specific short hairpin RNA (shRNA). The wound-healing and Transwell assays demonstrated that S100A6 promoted PDAC cell migration and invasion. Furthermore, β-catenin shRNA inhibited the migration and invasion of PDAC cells. We confirmed that S100A6 induces PDAC cell migration and invasion via activation of β-catenin in vitro. Assessment of mRNA and protein levels revealed that S100A6 induces increased expression of β-catenin, N-cadherin and vimentin, and decreased expression of E-cadherin in PDAC cells. β-catenin shRNA also altered the expression of epithelial-mesenchymal transition (EMT)-related markers in PDAC cells. Specifically, expression of E-cadherin was increased, whereas expression of N-cadherin and vimentin was decreased. Finally, we demonstrated that S100A6 alters the expression of EMT-related markers via β-catenin activation. In conclusion, S100A6 induces EMT and promotes cell migration and invasion in a β-catenin-dependent manner. S100A6 may therefore represent a novel potential therapeutic target for the treatment of pancreatic cancer.

## Introduction

Pancreatic ductal adenocarcinoma (PDAC) is a serious global health problem. It is the fourth most common cause of cancer-related death in the United States, with a 5-year overall relative survival rate of 6%[1]. In China, The median survival time of PDAC patients is 7.8 months, with 30.0% of patients undergoing curative intent operations, and only 9.8% of patients receiving comprehensive treatment[2]. Despite advances in treatment, PDAC remains extremely resistant to currently available radiotherapy and chemotherapy regimens[3]. One contributor to the poor prognosis is the limited understanding of the pathogenesis of pancreatic cancer. Therefore, there is an urgent need to elucidate the molecular mechanisms associated with the occurrence, development and metastasis of this lethal disease.

S100A6 belongs to the S100 family, expression of which is connected to tumorigenesis and metastasis[4]. Logsdon et al.[5] used microarrays to profile PDAC gene expression, identifying a total of 158 pancreatic cancer-related genes, including S100A6. Our group has previously performed immunohistochemical analysis of S100A6 expression in pancreatic tissues, confirming that S100A6 expression is elevated in PDAC samples, relative to normal tissues[6]. Ohuchida et al.[7] showed that expression of S100A6 is primarily restricted to the nuclei of pancreatic cancer cells, and high nuclear S100A6 protein expression levels are associated with a poor prognosis. The role of S100A6 in relation to tumor formation and metastasis is, however, poorly understood. Some studies have shown that S100A6 is involved in the regulation of the wnt/β-catenin signaling pathway[8], which leads to the degradation of β-catenin.

Wnt/β-catenin signaling influences cell fate, proliferation, polarity, and cell death during embryonic development, as well as tissue homeostasis in adults[9]. Aberrant regulation of this pathway is therefore associated with a variety of diseases, including cancer, fibrosis and neurodegeneration[10]. The wnt pathway is composed of the wnt ligand protein and cell surface receptor, in addition to cytoplasmic components and a specific nuclear transcriptional complex[11]. When the wnt ligand protein binds to frizzled, a cell surface receptor, the wnt pathway is activated. Cytoplasmic β-catenin then enters the cell nucleus, where it modulates transcription, thereby influencing cell proliferation and tumor metastasis. In this process, β-catenin is the key effector molecule[12]. A variety of cellular proteins, including wnt, can influence β-catenin production and accumulation in the cytoplasm. RNA sequencing of pancreatic circulating tumor cells implicates wnt and β-catenin in metastasis[13].

There is a wealth of research concerning the attributes of circulating tumor cells that relate to the epithelial-mesenchymal transition (EMT)[14,15]. EMT refers to the transdifferentiation of epithelial cells into mesenchymal cells under certain physiological and pathological conditions, accompanied by cell morphology and gene expression changes[16]. EMT occurs in a variety of processes, such as embryonic development, wound healing, some chronic diseases, and early stage tumor metastasis. Down-regulation of E-cadherin, an epithelial marker, is a hallmark of EMT. The loss of E-cadherin is accompanied by the upregulation of mesenchymal markers, such as N-cadherin and vimentin. EMT is necessary for the majority of tumor metastases, including PDAC[17]. The Wnt/β-catenin pathway is one of the most important signaling pathways involved in EMT induction.

In this report, we investigated the function and associated mechanism of S100A6 in relation to EMT induction, by assessing its influence on Panc-1 cell migration and invasion.

## Materials and Methods

### Cell Line

The human pancreatic cancer cell line, Panc-1, was purchased from the American Type Culture Collection (USA). Cells were maintained in Dulbecco’s Modified Eagle’s Medium (DMEM) (Invitrogen, USA) containing 10% fetal bovine serum (FBS, Invitrogen, USA), 1% penicillin and streptomycin (Invitrogen, USA) at 37°C with 5% CO_2_.

### Plasmids and RNA Interference

The complementary DNAs for human S100A6 and the negative control DNA were inserted into a GV146 plasmid vector purchased from Genechem (Shanghai, China). A shRNA sequence targeting β-catenin (5′-ATCACTGAGCCTGCCATCTGTGCTCTTCG-3′) and a negative control sequence were synthesized and ligated into the pRFP-C-RS vector (Origene Technologies, USA). The plasmids were amplified according to the manufacturer’s instructions. Sequence analysis after cloning revealed 100% homology to published sequences.

### Transient Transfection and Creation of Stable Cell Lines

The S100A6 overexpression plasmid and the control plasmid were transfected into Panc-1 cells using opti-MEM reduced-serum medium (Invitrogen, USA) and Lipofectamine 2000 (Invitrogen, USA), according to the manufacturer’s instructions.

β-catenin shRNA and control shRNA were transfected into Panc-1 cells as stated above. After transient transfection, Panc-1 cells were selected using 1.0 μg/mL puromycin, and maintained in growth medium supplemented with 1.0 μg/mL puromycin. Clonal populations of cells were isolated by transferring single clumps of cells into individual wells of a six-well plate. Cells were then maintained at 37°C with 5% CO_2_.

Then S100A6 overexpression and control plasmids were transfected into β-catenin knockdown stable cell lines, as stated above.

### Wound-healing Assay

Cells were seeded into six-well plates and transfected with the S100A6 overexpression or control plasmid, and β-catenin shRNA or control shRNA. After 36 h, complete medium was replaced with FBS-free DMEM, and after a further 6 h, the cell monolayers were wounded with a 200-μL sterile plastic tip. The cultures were thereafter maintained in FBS-free DMEM, and wound areas were observed and photographed using an inverted microscope at 0, 24 and 48 h after wounding. The wound width was measured using Image J software. Wound healing rate was assessed as follows:

$$Wound\;Healing\;Rate(\%)=\frac{0h\;width-24/48h\;width}{0h\;width}\times 100$$

### Transwell Assay

Transwell chambers (Corning, USA) with an 8-μm pore were used to assess migration in 24-well plates. After transfection, a 200-μL volume of cells, at a density of 1 × 10^5^/mL in FBS-free DMEM, was seeded into the upper chambers. The lower chambers were filled with DMEM containing 10% FBS as a stimulatory factor. The chambers were incubated in a humidified tissue culture incubator at 37°C, 5% CO_2_ atmosphere, for 8 h. Cells were then fixed with 95% ethanol for 20 min, stained by crystal violet for 30 min, and counted using a microscope with a 40× objective.

To assess cell invasion, BioCoat Matrigel (BD Biosciences) (300 μg/mL, 100 μL per chamber) was applied to the upper insert chambers 5 h before following the procedure for the migration assay described above.

### Quantitative Real-Time PCR

Total RNA from Panc-1 cells was isolated using TRIzol reagent (Invitrogen, USA). Total RNA was used as a template to synthesize cDNA, according to the manufacturer’s instructions (Invitrogen, USA). Real-time PCR was performed using a 7500 Real-Time PCR System (Applied Biosystems, USA), with a reaction mix consisting of 5-μL 2× SYBR Green Master Mix, 1-μL template, 0.4-μL forward primer, 0.4-μL reverse primer and 3.2-μL RNA-free water. The cycling conditions were 95°C for 10 min, followed by 40 cycles of 95˚C for 15 s and 60˚C for 1 min. Gene expression levels were normalized relative to that of GAPDH. All reactions were performed in triplicate. Relative mRNA levels were presented as 2^−ΔΔCt^[18]. The primers used are detailed in Table 1.

**Table 1:** Sequences of Primers for Real-time PCR.

Primer	Forward	Reverse
S100A6	AAGCTGCAGGATGCTGAAAT	CCCTTGAGGGCTTCATTGTA
β-catenin	TGCCAAGTGGGTGGTATAGAG	CGCTGGGTATCCTGATGTGC
E-cadherin	CGAGAGCTACACGTTCACGG	GGGTGTCGAGGGAAAAATAGG
N-cadherin	GGTTTGGAATGGGACAGTTC	CTTGAGCCTGAGACACGAT
Vimentin	GAACGCCAGATGCGTGAAATG	CCAGAGGGAGTGAATCCAGATTA
GAPDH	GGTATCGGAAGGACTC	GGATGATGTTCTGGAGAGC

### Western Blotting

After transfection, cells were harvested using RIPA lysis buffer (Solarbio, Beijing, China) supplemented with PMSF protease inhibitor (Solarbio, Beijing, China). The protein concentrations of the samples were determined using a protein quantitation kit (KeyGEN BioTECH, Nanjing, China). Samples (40–100 μg) were then subjected to 10% SDS-PAGE and transferred to a polyvinylidene difluoride membrane (PVDF). Membranes were blocked for 2 h in 5% nonfat dried milk. Membranes were incubated with specific antibodies at 4°C overnight. The following primary antibodies were used in this study: anti-β-catenin (Cell Signaling Technology 8480, USA), anti-E cadherin (Abcam ab1416, USA), anti-N cadherin (Abcam ab19348, USA), anti-vimentin (Abcam ab8069, USA) and anti-β-actin (Cell Signaling Technology 8457, USA). The membranes were then incubated with the corresponding fluorescent secondary antibodies (Odyssey). Western blots were quantified using Odyssey Infrared Imaging. Protein expression levels were normalized relative to that of β-actin.

### Statistical Analysis

All experiments were carried out in triplicate and repeated independently at least three times. Data are presented as means ± SD (standard deviation). Student’s *t*-test was used for statistical analysis. All data were analyzed using the SPSS 17.0 statistical software package and figures were generated using the GraphPad Prism 5 software. For all tests, p < 0.05 was considered to indicate statistical significance.

## Results

### S100A6 promotes PDAC cell migration and invasion in vitro

**Fig 1:**
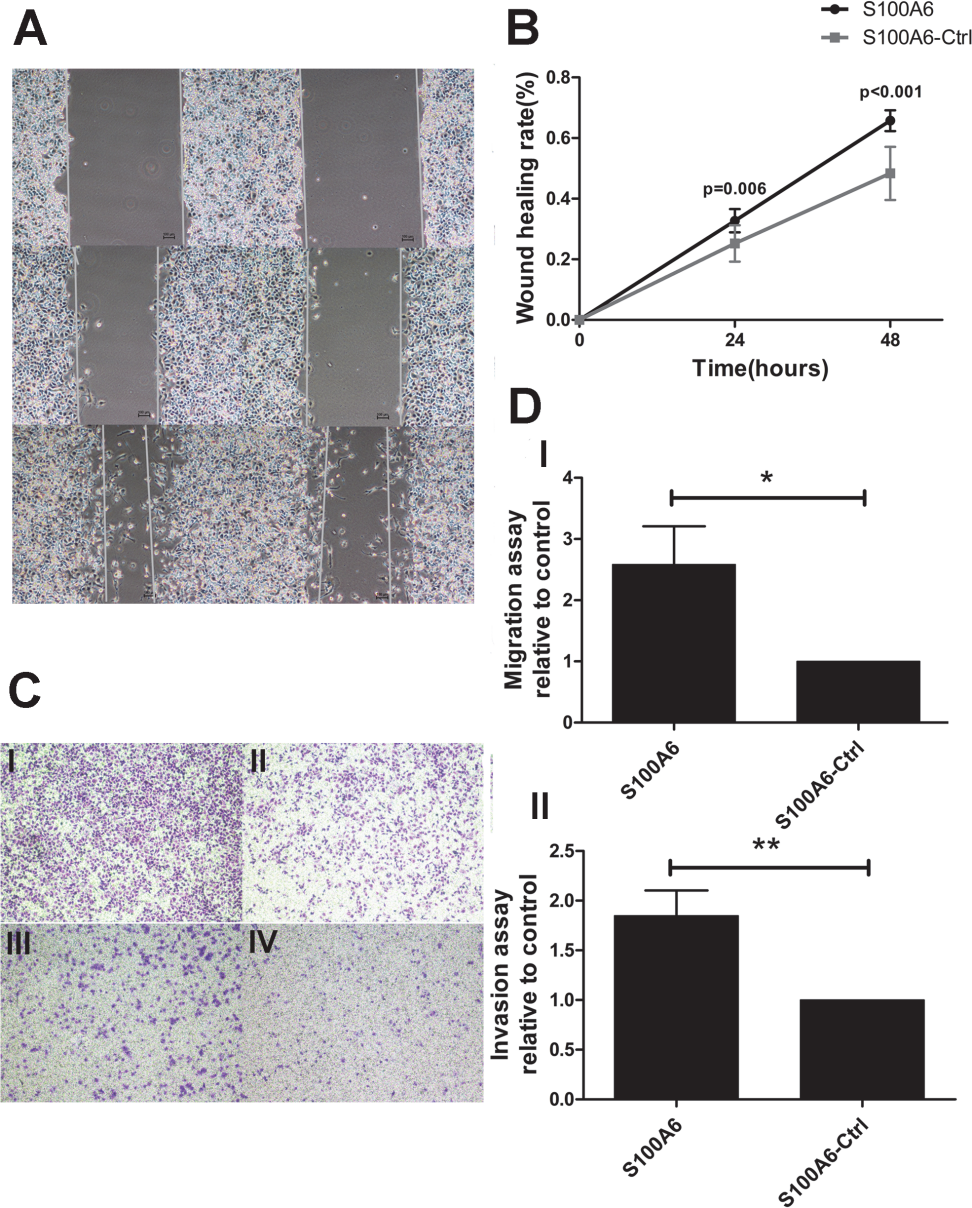
S100A6 promotes PDAC cell migration and invasion in vitro. (A) The wound-healing assay, cells overexpressing S100A6 (left), control cells (right). (B) The wound-healing rate was significantly higher in S100A6 overexpressing cells, relative to control cells, at 24 and 48 h. (C) Transwell assay, “I” represents S100A6 overexpression in the migration assay; “II” represents the control group in the migration assay; “III” represents S100A6 overexpression in the invasion assay; “IV” represents the control group in the invasion assay. (D) Representative statistical chart for the transwell assay, showing a significant increase in migration (I) and invasion (II) by S100A6 overexpressing cells, relative to control cells. *p < 0.05; **p < 0.01.

To assess whether S100A6 promotes PDAC cell migration and invasion, Panc-1 cells were transfected with a S100A6 overexpression plasmid or a control plasmid. Real-time RT-PCR was used to verify that the S100A6 overexpression plasmid increased the mRNA levels of S100A6 in Panc-1 cells. We detected a significant increase in the level of S100A6 mRNA in S100A6-overexpressing cells, compared to control cells (p < 0.001) (S1 Fig.). Next, we explored the effect of S100A6 expression on cell invasion in vitro. A wound-healing assay revealed a significant increase in wound healing rate in S100A6-overexpressing cells, compared to control cells, at 24 and 48 h (p = 0.006 and p < 0.001, respectively) (Fig. 1A and 1B). A transwell assay also demonstrated increased migration and invasion in S100A6 overexpressing cells (p = 0.04 for the migration assay, p = 0.006 for the invasion assay) (Fig. 1C and 1D). These data suggest that S100A6 promotes invasion of PDAC cells.

### S100A6 alters the expression of β-catenin and EMT-related markers in PDAC cells

**Fig 2:**
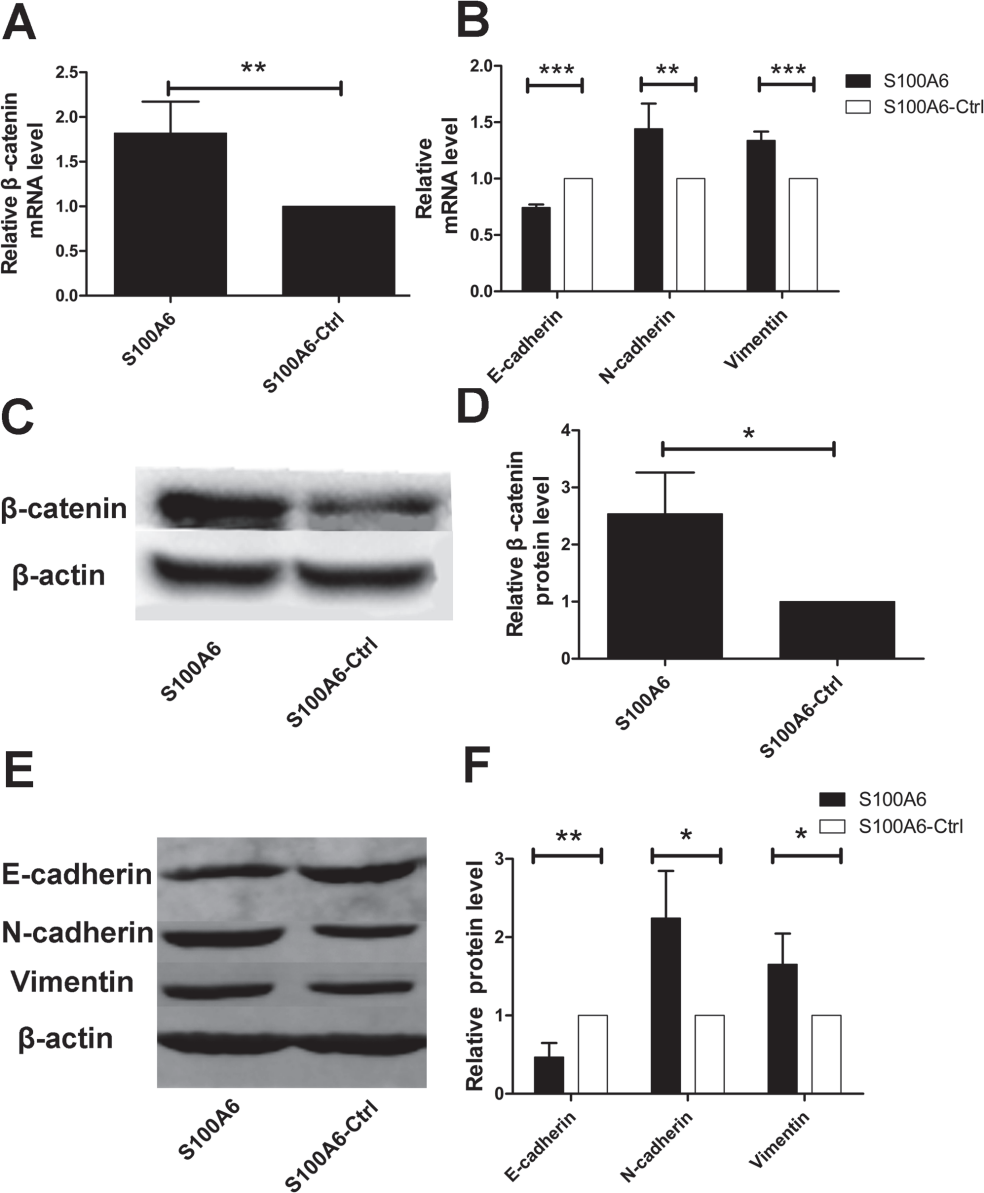
S100A6 overexpression alters the expression of β-catenin and EMT-related markers. (A) Overexpression of S100A6 resulted in a significant increase in the level of β-catenin mRNA in Panc-1 cells. (B) Real-time PCR revealed that S100A6 overexpression resulted in decreased expression of E-cadherin, and increased expression of N-cadherin and vimentin. (C) Western blotting revealed that S100A6 upregulated β-catenin on the protein level. (D) Representative statistical chart showing increased β-catenin protein levels, compared to the control. (E) Western blotting revealed that S100A6 overexpression resulted in altered expression of EMT-related markers. Specifically, protein expression of E-cadherin was decreased, whereas expression of N-cadherin and vimentin was increased. (F) The relative protein levels of E-cadherin, N-cadherin and vimentin differed significantly between the S100A6-overexpressing condition and the control. *p < 0.05; **p < 0.01; ***p < 0.001.

When investigating the possible downstream mediators of S100A6 responsible for promotion of PDAC cell migration and invasion, we noted that overexpression of S100A6 resulted in a significant increase in β-catenin mRNA levels in Panc-1 cells (p = 0.006) (Fig. 2A). Given that EMT plays an important role in the invasion-metastasis process, we proceeded to assess the expression of EMT-related markers. The expression of the epithelial marker E-cadherin was decreased at the mRNA level, while expression of the mesenchymal markers N-cadherin and Vimentin was increased (p < 0.001, p = 0.002, and p < 0.001, respectively) (Fig. 2B). We also examined the protein levels of β-catenin and the EMT markers using Western blotting, and confirmed that overexpression of S100A6 increased the expression of β-catenin (p = 0.024) (Fig. 2C and 2D), decreased that of E-cadherin, and increased the expression of N-cadherin and vimentin (p = 0.009, p = 0.026, p = 0.044, respectively) (Fig. 2E and 2F). Together, these results suggest that S100A6 elevates β-catenin levels and alters the expression of EMT-related markers in PDAC cells.

### β-catenin shRNA altered migration and invasion of PDAC cells in vitro

**Fig 3:**
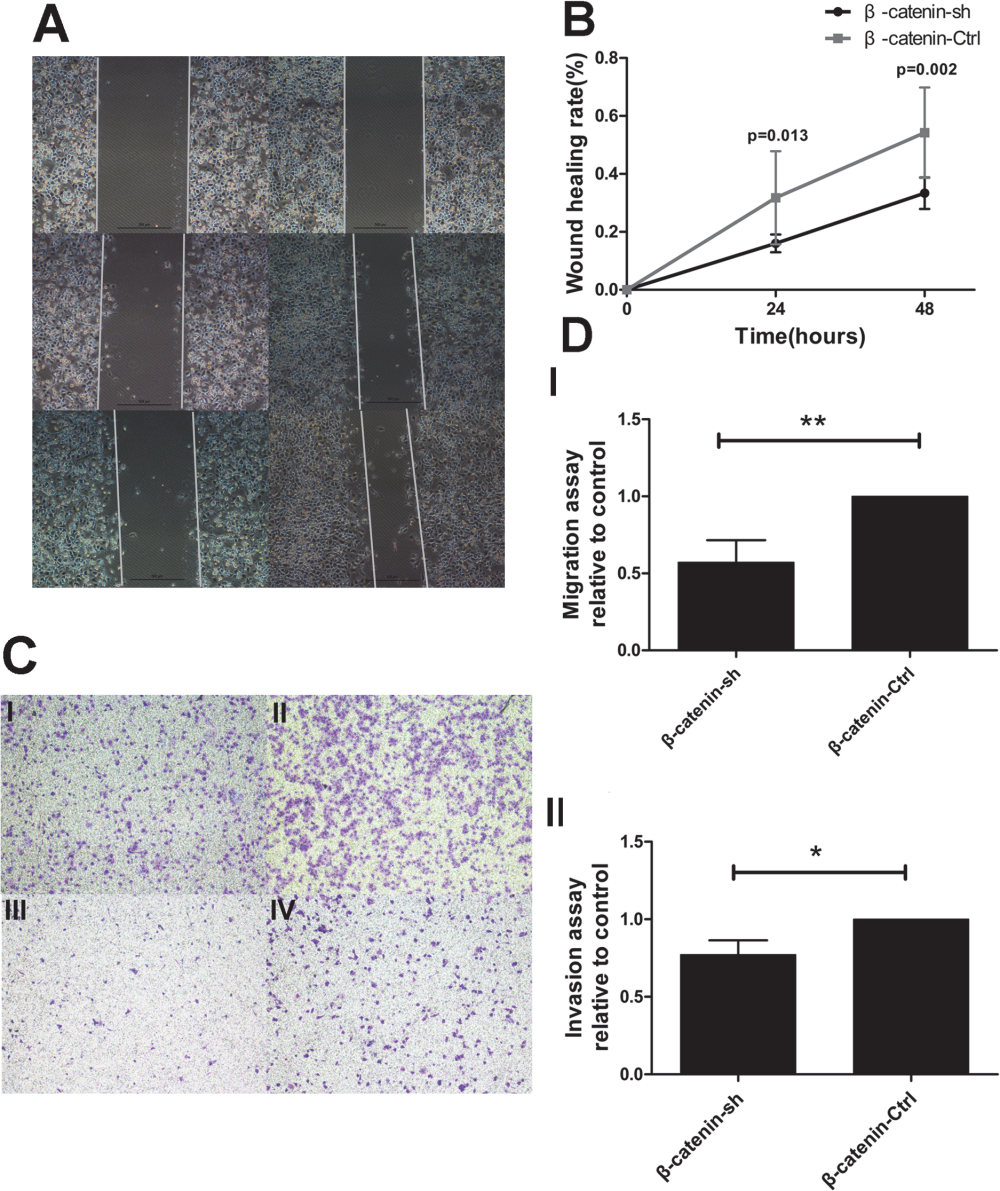
β-catenin shRNA alters the migration and invasion of PDAC cells in vitro. (A) The wound-healing assay revealed a significant decrease in migration in the β-catenin shRNA group (left), relative to the control group (right). (B) The wound-healing rate was significantly lower in S100A6-expressing cells, compared to the control, at 24 and 48 h. (C) The transwell assay demonstrated that β-catenin shRNA dramatically decreased the number of migrating and invading cells (×40). “I” represents β-catenin shRNA in the migration assay; “II” represents the control group in the migration assay; “III” represents β-catenin shRNA in the invasion assay; “IV” represents the control group in the invasion assay. (D) Representative statistical chart for the transwell assay, showing a significant decrease in migration (I) and invasion (II) in the β-catenin shRNA group, compared to the control group. *p < 0.05; **p < 0.01.

To explore the effect of β-catenin on migration and invasion of PDAC cells, we used β-catenin shRNA to generate a stable β-catenin knockdown Panc-1 cell line. Panc-1 cells transfected with non-target shRNA were used as a control. Knockdown of β-catenin was confirmed by real-time PCR and Western blotting (p < 0.001 and p = 0.001, respectively) (S2 Fig.). A wound-healing assay and transwell assay were carried out to assess the effect of β-catenin on migration and invasion. The wound-healing assay demonstrated that knockdown of β-catenin significantly inhibited the migration of PDAC cells compared to the control condition at both 24 and 48 h (p = 0.013 and p = 0.002, respectively) (Fig. 3A and 3B). The transwell assay revealed decreased migration and invasion in the β-catenin knockdown cells, compared to the control (p = 0.009 for the migration assay, p = 0.015 for the invasion assay) (Fig. 3C and 3D). These data indicate that inhibition of β-catenin reduces the migration and invasion of PDAC cells in vitro.

### β-catenin shRNA alters the expression of EMT-related markers in PDAC cells in vitro

**Fig 4:**
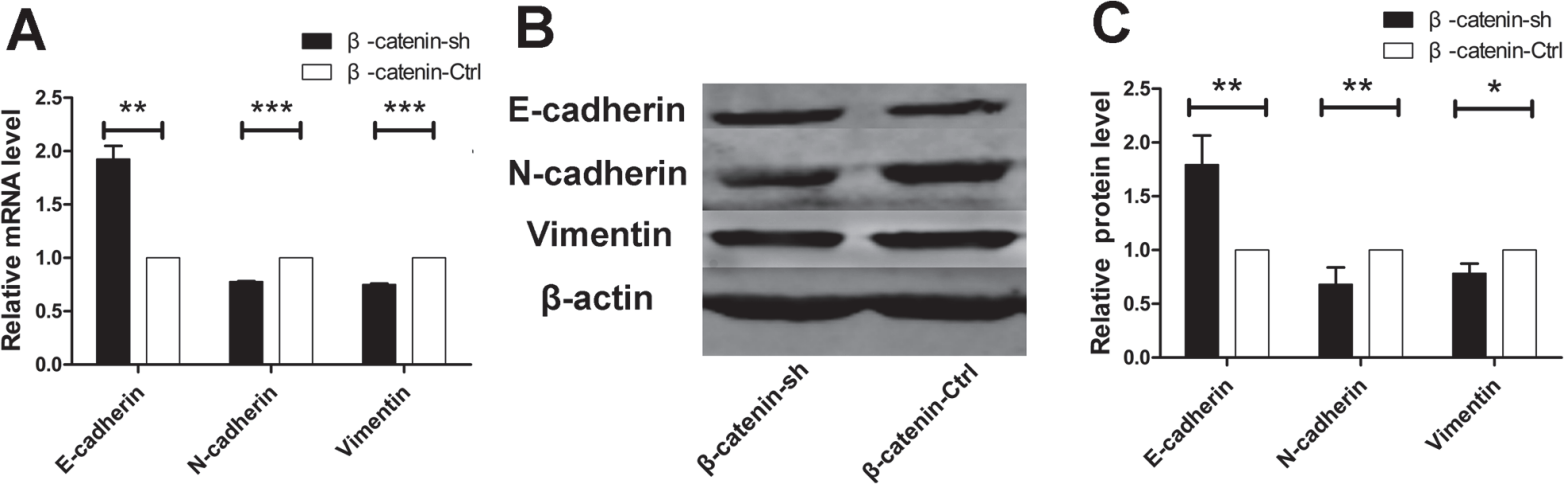
β-catenin shRNA alters the expression of EMT-related markers in PDAC cells in vitro. (A) β-catenin shRNA resulted in a significant increase in the levels of E-cadherin mRNA, and a significant decrease in the levels of N-cadherin and vimentin mRNA. (B) Western blotting revealed that β-catenin shRNA resulted in upregulated protein expression of E-cadherin and downregulated protein expression of N-cadherin and vimentin. (C) The protein levels of E-cadherin, N-cadherin and vimentin were significantly different between the β-catenin shRNA group and the control group. *p < 0.05; **p < 0.01; ***p < 0.001.

To evaluate whether β-catenin is involved in the EMT, we assessed the expression of EMT-related markers at the mRNA and protein levels in β-catenin-knockdown Panc-1 cells. Knockdown of β-catenin resulted in increased levels of E-cadherin mRNA, and decreased levels of N-cadherin and vimentin mRNA (p = 0.002, p < 0.001, and p < 0.001, respectively) (Fig. 4A). Western blotting results concerning protein expression were consistent with the real-time RT-PCR data. Specifically, protein expression of E-cadherin was increased, while expression of N-cadherin and vimentin was decreased (p = 0.004, p = 0.007, and p = 0.017, respectively), in β-catenin-knockdown Panc-1 cells (Fig. 4B and 4C). These data suggest that the β-catenin expression level correlates with the expression of EMT-related markers in PDAC cells.

### S100A6 promotes PDAC cell migration and invasion via β-catenin activation in vitro

**Fig 5:**
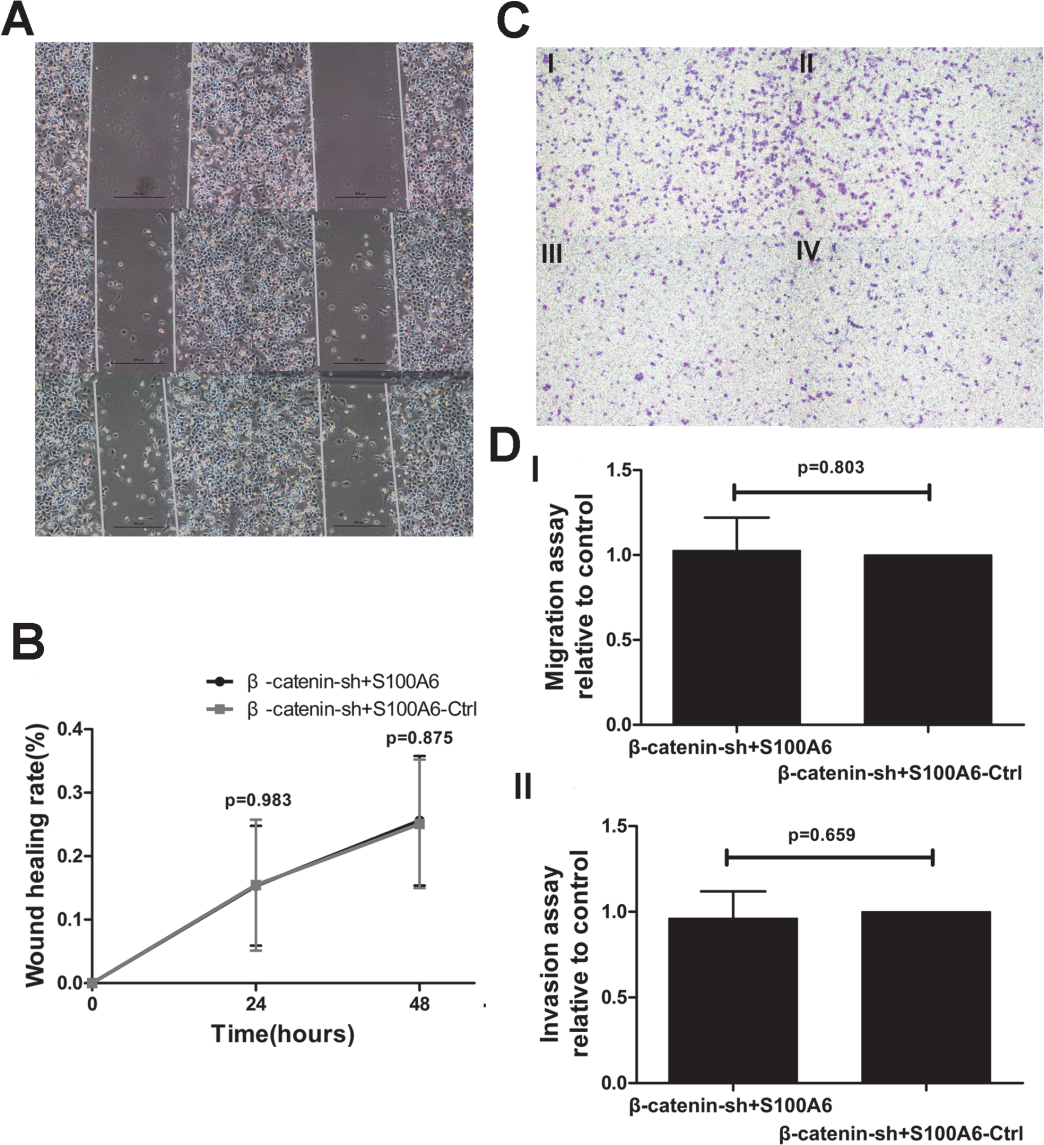
S100A6 overexpression did not influence migration and invasion in β-catenin-knockdown stable cells. (A) The wound-healing assay revealed no change in the migration of β-catenin-shRNA cells overexpressing S100A6 (left) compared to the β-catenin-shRNA control group (right). (B) There was no significant difference in the wound-healing rate between the two groups. (C) There was no significant difference in the transwell assay results between the two groups, with respect to both migration and invasion (×40). “I” represents β-catenin-shRNA+S100A6 overexpression in the migration assay; “II” represents β-catenin-shRNA+S100A6-control in the migration assay; “III” represents β-catenin-shRNA+S100A6 overexpression in the invasion assay; “IV” represents β-catenin-shRNA+S100A6-control in the invasion assay. (D) Representative statistical chart for the transwell assay, showing no significant difference in the migration (I) or invasion (II) assays between the two groups.

To explore whether S100A6 promotes PDAC cell migration and invasion via regulation of β-catenin, we transfected stable β-catenin-knockdown Panc-1 cells with a S100A6 overexpression plasmid. We assessed β-catenin expression using real-time RT-PCR and Western blot analyses. There were no significant differences in the mRNA and protein levels between the two groups (p = 0.354 and p = 0.765, respectively) (S3 Fig.). These results suggest that β-catenin was indeed stably knocked down, and that its expression was not influenced by S100A6 overexpression. Next, we performed wound-healing and transwell assays to assess migration and invasion of these cells. The wound-healing assay demonstrated that migration was not significantly different between the two groups, at 24 or 48 h (p = 0.983 and p = 0.875, respectively) (Fig. 5A and 5B). The results of the transwell assay were consistent with the wound-healing assay, with no significant difference between the groups for either migration or invasion (p = 0.803 for the migration assay, p = 0.659 for the invasion assay) (Fig. 5C and 5D). These data indicate that S100A6 has no effect on migration and invasion in stable β-catenin-knockdown PDAC cells. This suggests that S100A6 promotes PDAC cell migration and invasion via β-catenin activation in vitro.

### S100A6 promotes EMT through activation of β-catenin

**Fig 6:**
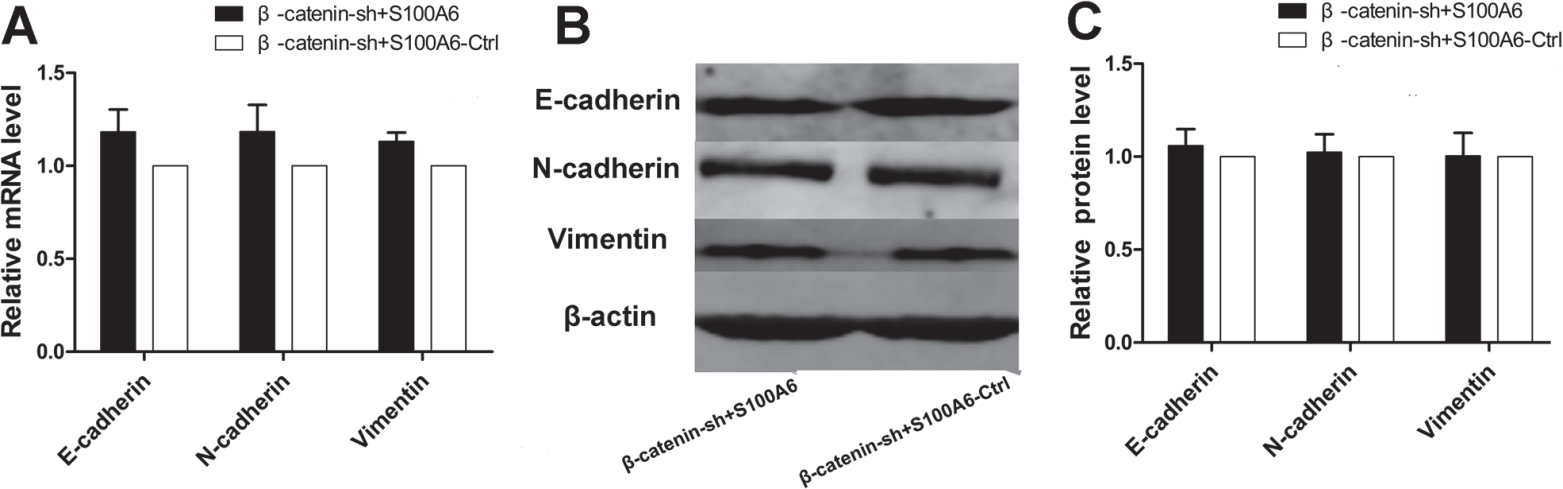
S100A6 overexpression did not alter the expression of EMT-related markers in β-catenin-knockdown stable cells. (A) There were no significant differences in the expression of EMT-related markers between β-catenin-shRNA cells overexpressing S100A6 and β-catenin-shRNA control cells. (B) Western blotting revealed similar changes in the expression of EMT-related markers in the two groups. (C) No significant differences were seen between the two groups with respect to the expression of E-cadherin, N-cadherin and vimentin protein.

To investigate the potential mechanistic relationship between S100A6 and β-catenin in relation to EMT in pancreatic cancer, we transfected stable β-catenin-knockdown Panc-1 cells with a S100A6 overexpression plasmid or a control plasmid, and evaluated the expression of EMT-related markers. Real-time RT-PCR demonstrated no significant differences in the mRNA levels of E-cadherin, N-cadherin and vimentin between the two groups (p = 0.227, p = 0.228, and p = 0.067, respectively) (Fig. 6A). We confirmed that the protein levels of E-cadherin, N-cadherin and vimentin also did not differ between the two groups (p = 0.267, p = 0.638, and p = 0.940, respectively) (Fig. 6B and 6C). These results suggest that S100A6 has no effect on EMT-related markers in stable β-catenin-knockdown PDAC cells, indicating that S100A6 alters the expression of EMT-related markers via activation of β-catenin.

## Discussion

In this study, we demonstrated that S100A6 overexpression promotes, and β-catenin shRNA inhibits, PDAC cell migration and invasion. We established that S100A6 promotes PDAC cell migration and invasion via β-catenin activation in vitro. We also confirmed that S100A6 expression correlates with that of β-catenin and that the expression of EMT-related markers in PDAC cells is modified by both S100A6 overexpression and β-catenin knockdown.

S100A6 is reported to be upregulated in a number of cancers, including lung, colorectal, skin, gastric, pancreatic ductal adenocarcinoma and other epithelial cancers[19]. It is reported that increased expression of S100A6 can promote cell proliferation and migration in human hepatocellular carcinoma[20]. Moreover, serum S100A6 levels may serve as a potential prognostic biomarker in gastric cancer, and inhibition of S100A6 decreases the metastatic potential of gastric cancer cells[21]. In pancreatic carcinogenesis, immunohistochemistry assays have been used to demonstrate that PanIN 1a cells do not express S100A6, and that the proportion of positively stained cells increases proportionately with the PanIN grade, with S100A6 mRNA levels also increasing in a stepwise manner[22,23].

A wide range of experimental models are suggestive of an important role for β-catenin in pancreatic cancer metastasis[24]. Nowotny et al.[25,26] suggested that S100A6 might have an effect on calcyclin-binding protein (CacyBP) /Siah-1 interacting protein (SIP) and suppressor of G2 allele of Skp1 (Sgt1). CacyBP/SIP was identified as a S100A6-binding protein that is involved in the ubiquitination and degradation of β-catenin[27]. The sequence of Sgt1 shares 20% identity with that of CacyBP/SIP. Furthermore, Sgt1 and CacyBP/SIP were found to bind Skp1 and activate Skp1p-cullin-F-box protein (SCF) ubiquitin ligase. This ligase is described in mammalian cells as a complex that regulates the ubiquitination and degradation of phosphorylated β-catenin[28]. Another study[29] confirmed that CacyBP/SIP is not detected in normal pancreatic tissues but is detected in pancreatic cancer. We therefore speculate that S100A6 affects the degradation of β-catenin via Cacy BP/SIP and Sgt1 in PDAC. In this study, S100A6 induced β-catenin expression at both the mRNA and protein levels. Moreover, both a wound-healing assay and a transwell assay demonstrated that S100A6 overexpression promotes PDAC cell migration and invasion.

In numerous models, including mammary epithelial and carcinoma cell lines, the Wnt/β-catenin pathway is thought to induce EMT. β-catenin is usually bound to E-cadherin, which is important for cell-cell connections and the cadherin cytoskeleton. In addition, β-catenin transportation into the nucleus can promote the expression of EMT related genes[30]. Some studies[31–34] have confirmed that certain factors can control epithelial plasticity and alter metastasis through β-catenin-dependent regulation in PDAC, colorectal cancer, hepatocellular carcinoma and breast cancer. Our study also demonstrated that β-catenin knockdown can induce upregulation of E-cadherin and downregulation of N-cadherin and vimentin.

A recent study reported that S100A4, a member of the S100 family, could be a key factor in promoting the EMT process in PDAC[35]. In this study, we explored the relationships among S100A6, β-catenin, EMT and PDAC cell invasion. We confirmed that S100A6 might induce EMT in a β-catenin-dependent manner, by demonstrating that S100A6 can promote cell migration and invasion through β-catenin in vitro. The exact role of the S100 family and related proteins with respect to pancreatic cancer is an important issue that merits further study.

## Conclusion

S100A6 can induce EMT and promote cell migration and invasion in a β-catenin-dependent manner. S100A6 may therefore represent a novel potential therapeutic target for PDAC.

## Supporting Information

S1 Fig Transfection with the S100A6 overexpression resulted in a significant increase in the S100A6 mRNA level. ***p < 0.001.
(TIF)

S2 Fig β-catenin was successfully knocked down in PDAC cells. (A) Real-time RT-PCR revealed a significant decrease in the β-catenin mRNA level in β-catenin shRNA Panc-1 cells. (B) Western blotting revealed a significant decrease in β-catenin protein in the β-catenin shRNA group, relative to the control. (C) The level of β-catenin protein was significantly different between the two groups. **p < 0.01; ***p < 0.001.
(TIF)

S3 Fig β-catenin expression was not upregulated by S100A6 in β-catenin-knockdown stable cells. (A) There were no significant differences in β-catenin mRNA levels between stable β-catenin-knockdown cells overexpressing S100A6 and stable β-catenin knockdown cells expressing the control plasmid. (B) Western blotting revealed similar changes in β-catenin between the two groups. (C) There were no significant differences between the β-catenin protein levels between the two groups.
The English in this document has been checked by at least two professional editors, both native speakers of English. For a certificate, please see:http://www.textcheck.com/certificate/GZTsUk
(TIF)
