# Supplementary figures and images for: S100 Calcium-Binding Protein A6 Promotes Epithelial-Mesenchymal Transition through β-Catenin in Pancreatic Cancer Cell Line
Source: PLoS One. 2015 Mar 23;10(3):e0121319. doi: 10.1371/journal.pone.0121319 (PMC4370615; doi:10.1371/journal.pone.0121319)

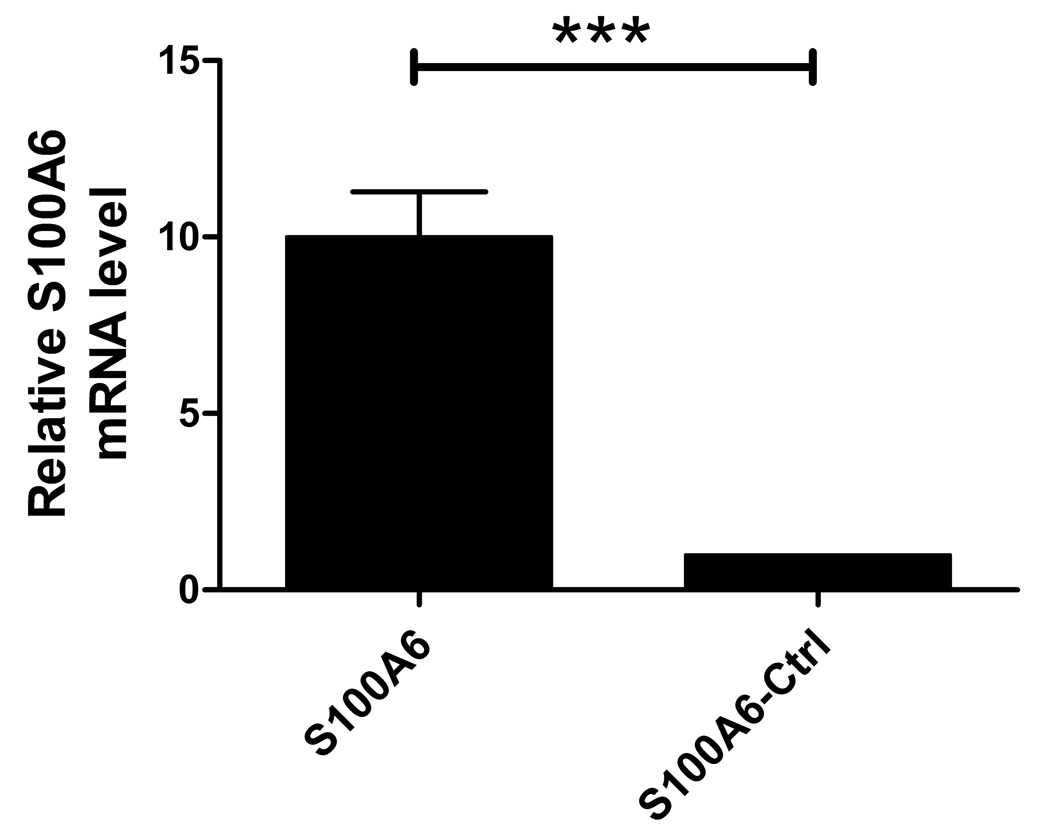

Supplement: S1 Fig — ***p < 0.001. (TIF) [file pone.0121319.s001.tif]

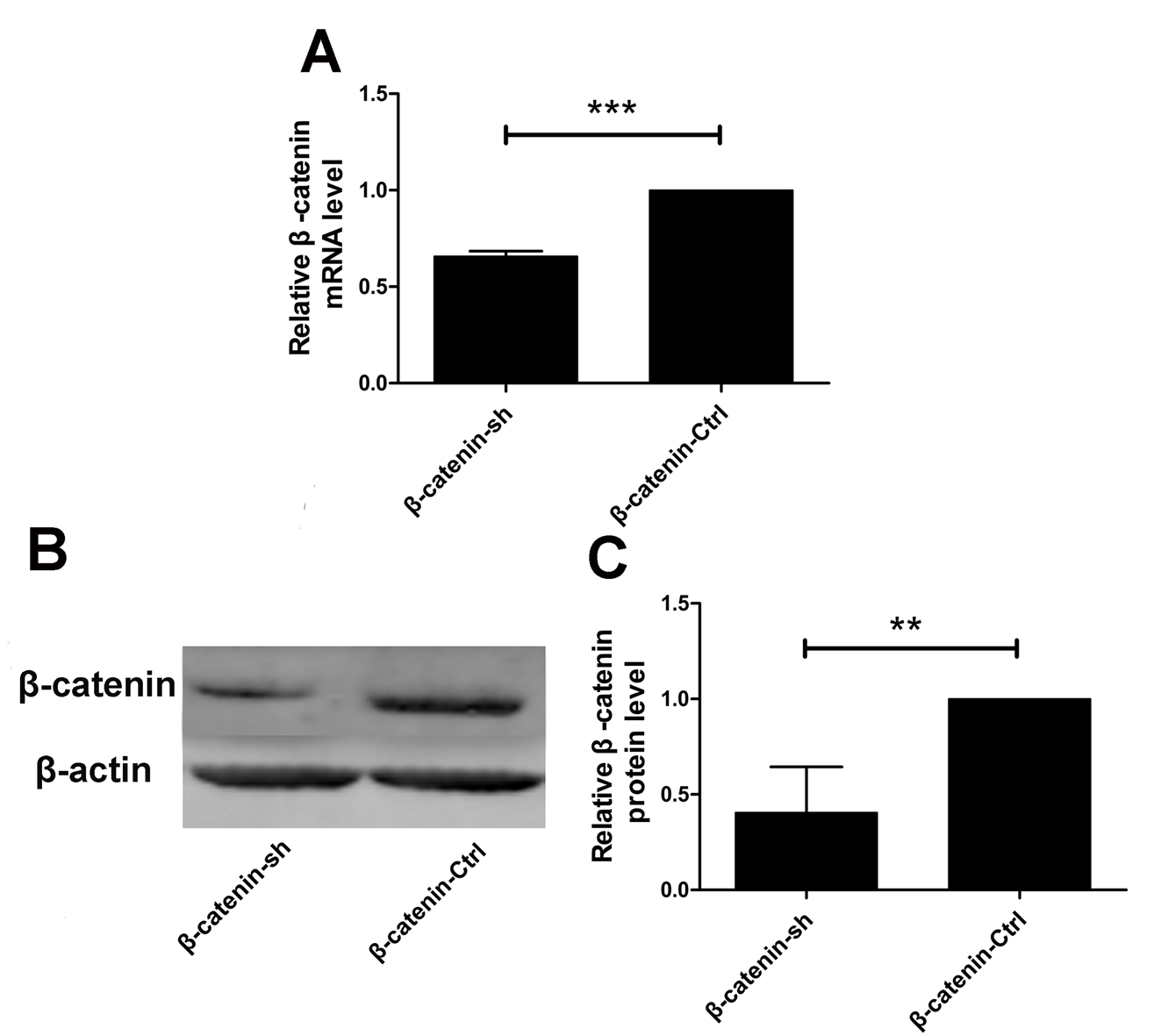

Supplement: S2 Fig — (A) Real-time RT-PCR revealed a significant decrease in the β-catenin mRNA level in β-catenin shRNA Panc-1 cells. (B) Western blotting revealed a significant decrease in β-catenin protein in the β-catenin shRNA group, relative to the control. (C) The level of β-catenin protein was significantly different between the two groups. **p < 0.01; ***p < 0.001. (TIF) [file pone.0121319.s002.tif]

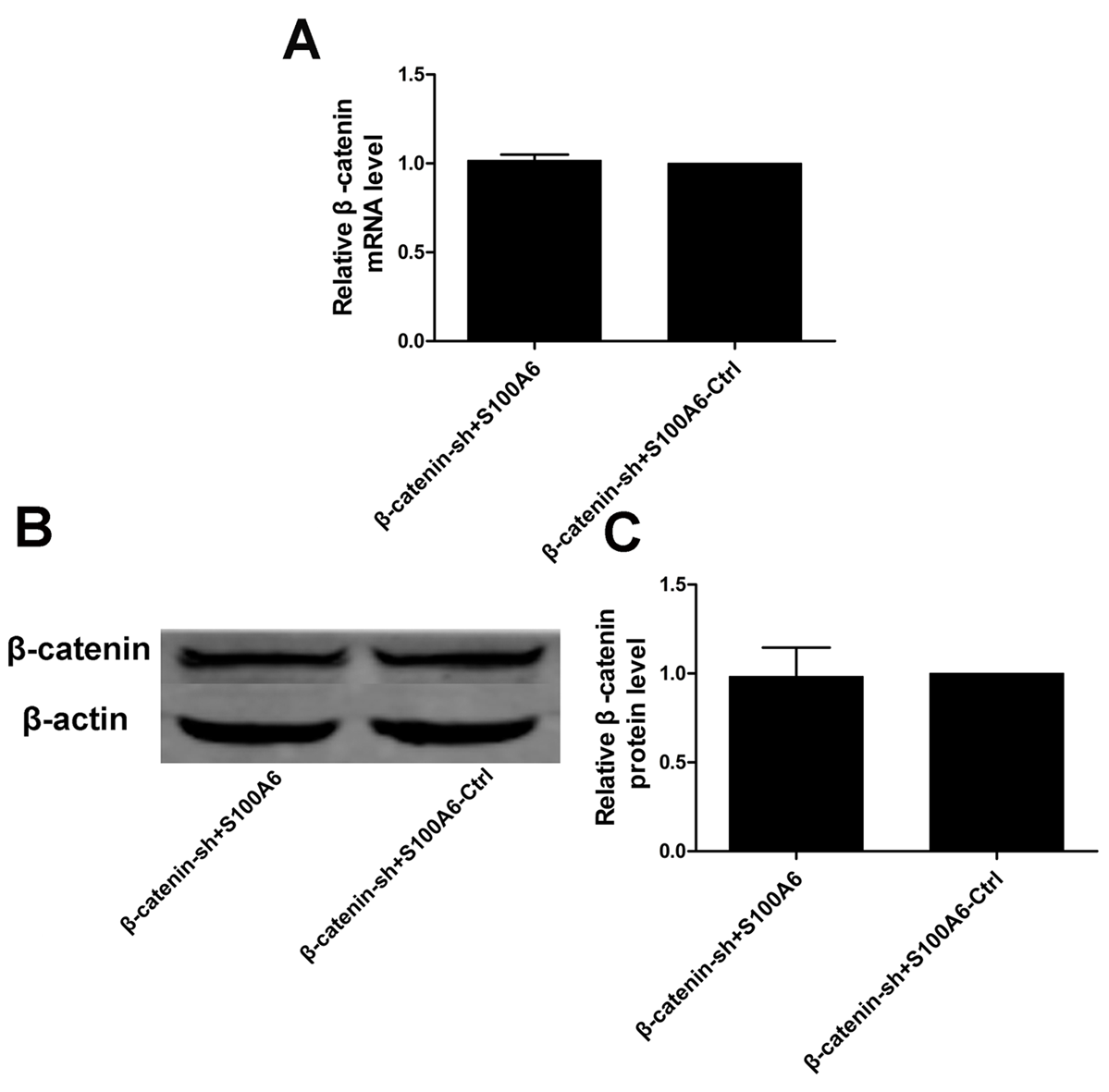

Supplement: S3 Fig — (A) There were no significant differences in β-catenin mRNA levels between stable β-catenin-knockdown cells overexpressing S100A6 and stable β-catenin knockdown cells expressing the control plasmid. (B) Western blotting revealed similar changes in β-catenin between the two groups. (C) There were no significant differences between the β-catenin protein levels between the two groups. The English in this document has been checked by at least two professional editors, both native speakers of English. For a certificate, please see:http://www.textcheck.com/certificate/GZTsUk (TIF) [file pone.0121319.s003.tif]
